# Visualization of Endolymphatic Hydrops in Patients With Unilateral Idiopathic Sudden Sensorineural Hearing Loss With Four Types According to Chinese Criterion

**DOI:** 10.3389/fsurg.2021.682245

**Published:** 2021-06-21

**Authors:** Huan Qin, Baihui He, Hui Wu, Yue Li, Jianyong Chen, Wei Wang, Fan Zhang, Maoli Duan, Jun Yang

**Affiliations:** ^1^Department of Otorhinolaryngology Head and Neck Surgery, Xinhua Hospital, Shanghai Jiaotong University School of Medicine, Shanghai, China; ^2^Ear Institute, Shanghai Jiaotong University School of Medicine, Shanghai, China; ^3^Shanghai Key Laboratory of Translational Medicine on Ear and Nose Diseases, Shanghai, China; ^4^Department of Otolaryngology Head and Neck, Audiology and Neurotology, Karolinska University Hospital, Stockholm, Sweden; ^5^Division of Ear, Nose and Throat Diseases, Department of Clinical Science, Intervention and Technology, Karolinska Institute, Stockholm, Sweden

**Keywords:** idiopathic sudden sensorineural hearing loss, endolymphatic hydrops, low-frequency hearing loss, magnetic resonance imaging, prognosis

## Abstract

**Objective:** The aim of this study is to evaluate the possible value of endolymphatic hydrops (EH) in patients with unilateral idiopathic sudden sensorineural hearing loss (UISSNHL) with four types according to audiometry.

**Methods:** Seventy-two patients (40 men and 32 women; age range, 28–78 years; mean age: 50.0 ± 12.9 years) with UISSNHL were admitted retrospectively into this study. Based on the pure tone audiometry before treatment, the hearing loss of all these patients were categorized into four types: low-frequency group (LF-G), high-frequency group (HF-G), flat group (F-G), and total deafness group (TD-G). The average time from symptom onset to the first examination was 6.9 ± 4.4 days (1–20 days). 3D-FLAIR MRI was performed 24 h after intratympanic injection of gadolinium (Gd) within 1 week after the UISSNHL onset. The incidence of EH in the affected ears based on four types of hearing loss were analyzed using the Chi-square test, and the possible relationship with vertigo and prognosis were also assessed.

**Results:** Eleven of 21 patients (52.4%) in LF-G had the highest EH-positive rate, followed by 18.2% in HF-G, 11.8% in F-G, and 17.4% in TD-G. The significant difference was found in the four groups (*P* = 0.018). The EH rate of LF-G was statistically significantly higher than that of F-G and TD-G (*P* = 0.009, *P* =0.014), respectively. After being valued by the volume-referencing grading system (VR scores), the EH level was represented by the sum scores of EH. In LF-G, no statistically significant difference was found in the prognosis of ISSNHL patients between with the EH group and the no EH group (*P* = 0.586). The symptom “vertigo” did not correlate with EH and prognosis.

**Conclusions:** EH was observed in UISSNHL patients by 3D-FLAIR MRI. EH may be responsible for the pathology of LF-G but not related to prognosis. It might be meaningless to assess EH in other hearing loss types, which might be more related to the blood-labyrinth dysfunction.

## Introduction

Sudden sensorineural hearing loss (SSNHL) is defined as a subset of disorder in which hearing loss is sensorineural and occurs within 72 h, affecting ~5–27 per 100,000 people annually (1). Furthermore, SSNHL is one of the most frequently recognized otolaryngological emergencies (2). About 90% of patients with SSNHL have no identifiable cause for the hearing loss (1). Rather than the possible tumor, trauma, or other causes identifiable according to patients' history (2), the exact etiology and pathological mechanism of idiopathic sudden sensorineural hearing loss (ISSNHL) have not been clarified (3). Hypothesis causes of ISSNHL mainly focused on micro-circulation disorders, viral infection, or autoimmune diseases (3). However, the possible relationship between EH and hearing loss was also mentioned early in 2002 (4); however, few articles published to support due to the limited imaging techniques.

The study of the correlation of EH with ISSNHL started prospering not only because the imaging of EH was successfully settled by the three-dimensional fluid-attenuated inversion recovery (3D-FLAIR) magnetic resonance imaging (MRI) by Nagawama et al. (5) and Nakashima et al. (6) but also due to the progressive understanding of EH in Ménière's disease (MD). Foster and Breeze (7) concluded EH as a cofactor of MD with other possible stimuli like ischemia. Venous drainage had an influence on both ISSNHL and MD reported in 2008, also suggesting the possible relationship between EH and ISSNHL (8). Chen et al. (9) reported that EH was observed in four of seven ISSNHL patients with vertigo. Okazaki et al. (10) found that cochlear EH and vestibular EH were observed in 66 and 41% of the affected ears with ISSNHL, respectively. Zheng et al. (11) claimed that the presence of EH may be a secondary reaction following the impairment of the inner ears with pantonal ISSNHL (a German classification in which the hearing level at all frequencies decreases to the approximate degree between 35 and 120 dB) because no correlation between vertigo and prognosis was found.

In the present study, 3D-FLAIR MRI of membranous labyrinth after intratympanic gadolinium (Gd) injection was performed and EH was assessed according to the volume-referencing grading system (VR scores) proposed in our previous study (12). The EH was analyzed in four types of unilateral ISSNHL (UISSNHL) according to a Chinese guideline published in 2015 (13), which is adjusted from the classification standard proposed by Sheehy (14) and mentioned in a Chinese multicenter study (15). The purpose of this study is to find the EH distribution in four types of UISSNHL and to preliminarily explore the possible value of EH among different types.

## Materials and Methods

### Subjects

In this retrospective study, 72 patients (40 men and 32 women; age range, 28–78 years; mean age: 50.0 ± 12.9 years) were enrolled from March 2017 to June 2020 in the Department of Otolaryngology-Head and Neck Surgery, Xinhua Hospital, Shanghai Jiaotong University School of Medicine. All patients met the following inclusion criteria; the first three criteria were from the 2015 Chinese guideline (Table 1) (13): (1) patients had experienced a UISSNHL and had an audiometry within 72 h; (2) the cause of hearing loss was unclear after detailed history collection; (3) the extent of hearing loss was at least 20 dB HL in at least two contiguous frequencies with no air-bone conduction gap [compared to the 2019 AAO-HNS criteria (1): hearing loss consists of a decrease in hearing of 30 dB HL affecting at least three consecutive frequencies]; and (4) 3D-FLAIR MRI was done within 1 week of the onset of hearing loss. Exclusion criteria included were the following: (1) a history of (fluctuating or acute) sensorineural hearing loss; (2) patients with vertigo caused by benign paroxysmal positional vertigo, MD, and vestibular schwannoma; (3) a history of previous otologic surgery, middle ear disease, cranial disease, or head trauma; (4) otalgia in bilateral ears; and (5) an allergy to Gd. Before detailed history collection, MRI examination, and treatment, the informed consent was obtained from each participant. The information included age, sex, affected side were collected.

**Table 1:** Criteria for diagnosis of ISSNHL.

**Main symptoms**
Sudden onset
Sensorineural hearing loss
Unknown etiology
**For reference**
Hearing loss ≥ 20 dB HL in at least two adjacent frequencies occurred suddenly within 72 h
Mostly unilateral hearing loss, few bilateral onset occurred simultaneously or successively
May be accompanied by tinnitus, stuffy feeling of ear, abnormal feeling of skin around the ear
May be accompanied by vertigo, nausea, and vomiting
No cranial nerve symptoms other than from cranial nerve VIII
Definite diagnosis: all of the above main symptoms are present

*These criteria were assessed by the Society of Otorhinolaryngology Head and Neck Surgery Chinese Medical Association of China in 2015*.

### Pure-Tone Audiometry

**Figure 1:**
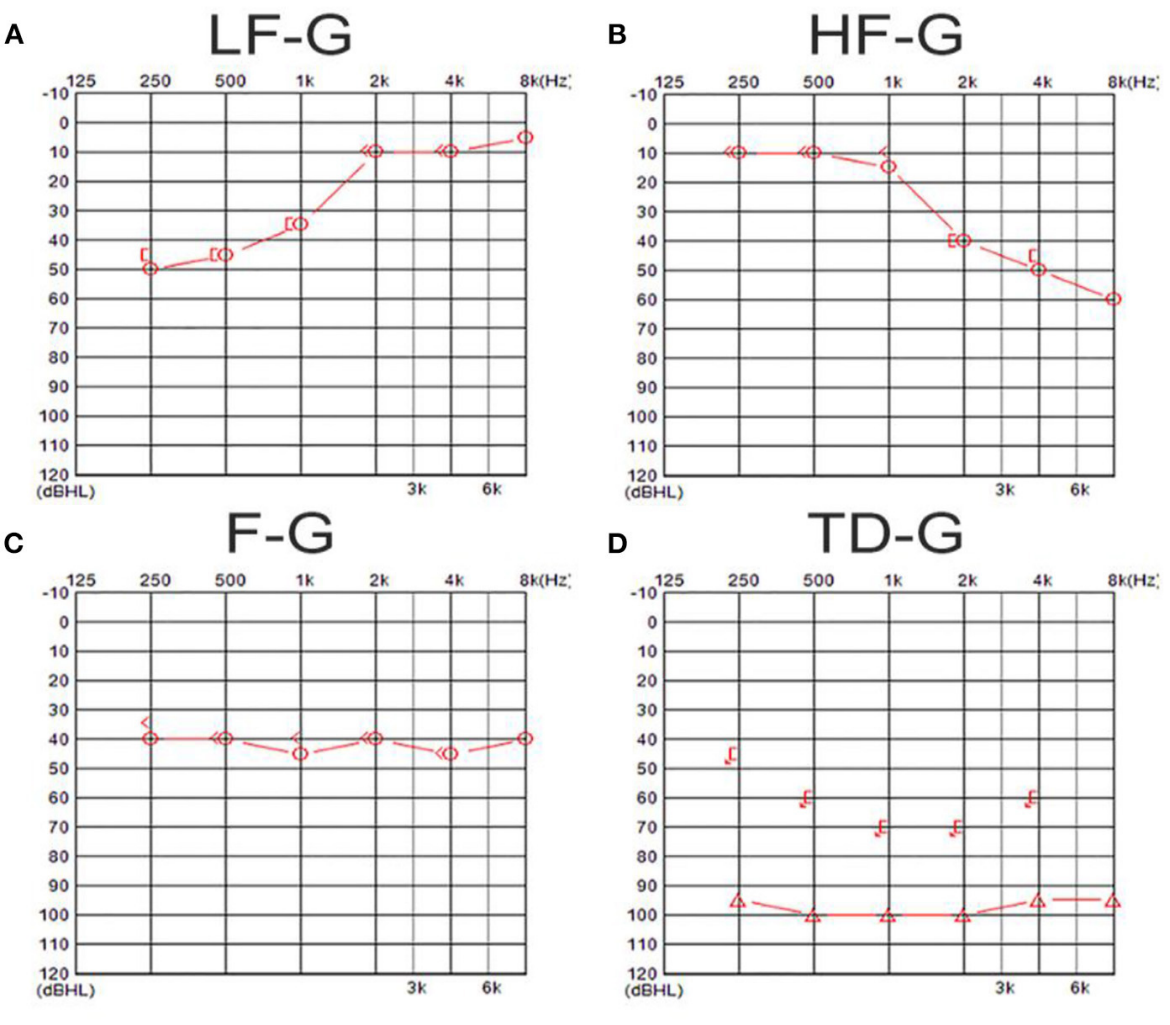
The examples of audiograms in four types of UISSNHL. **(A)** Shows an audiogram of a patient in LF-G in which hearing loss was over 20 dB HL in 250–1 k-Hz frequencies; **(B)** shows an audiogram of a patient in HF-G in which hearing loss was over 20 dB HL in 2–8 k-Hz frequencies; **(C)** exhibits an audiogram of a patient in F-G in which hearing loss was at all frequencies and the average hearing threshold was ≤80 dB HL; **(D)** shows an audiogram belonging to a patient in TD-G in which hearing loss at all frequencies was ≥81 dB HL.

Pure-tone audiometry was performed in a soundproof room with the use of an audiometer (Type Madsen, Astera, München, Denmark). According to the 2015 Chinese guideline (13), the diagnosis of UISSNHL was established and divided into four groups: (1) low-frequency group (LF-G): hearing loss at frequencies under 1 k Hz with the least reduction by 20 dB HL at 250 and 500 Hz; (2) high-frequency group (HF-G): hearing loss at frequencies of 2 k Hz or above with the least reduction by 20 dB HL at 4 and 8 k Hz; (3) flat group (F-G): hearing loss at all frequencies (250–8 k Hz) and the average hearing threshold was ≤ 80 dB HL; and (4) total deafness group (TD-G): hearing loss at all frequencies (250–8 k Hz) with the mean threshold ≥81 dB HL. The examples of audiograms in four types are shown in Figure 1. The audiometry was collected both before and 1 month after the treatment. The pure tone average (PTA) was calculated as the average threshold of those damaged frequencies: 250–1 k Hz in LF-G; 2–8 k Hz in HF-G; and 250–8 k Hz in F-G and TD-G.

### Intratympanic Gd Injection and MRI Analysis

Gd was diluted eight-fold with saline (v/v = 1:7) and injected intra-tympanically (0.5 ml) through the inferior–posterior quadrant of the tympanic membrane bilaterally using a 23-G needle and a 1-ml syringe under an oto-endoscope. The patient was then placed in the supine position for 60 min.

**Figure 2:**
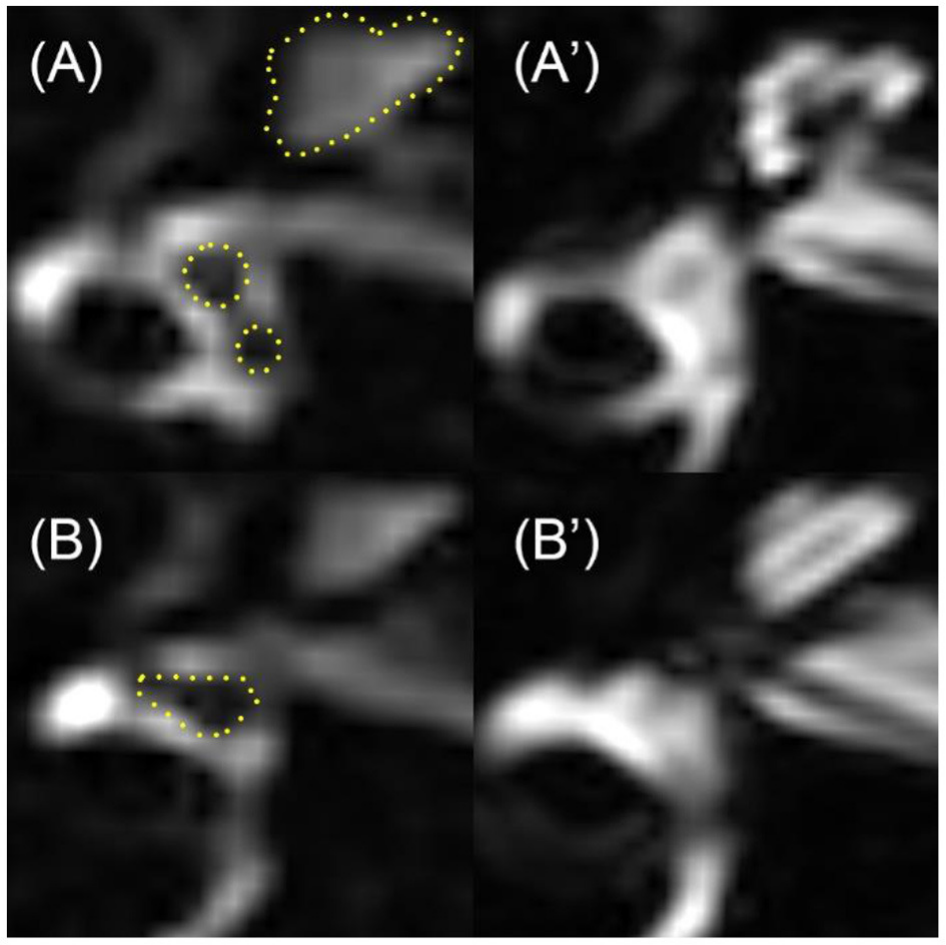
An example of the EH in an UISSNHL patient. This patient belonged to LF-G. 3D-FLAIR images on the left were compared with T2 images on the right. The evaluation was as follows: cochlea-grade I (three points). The cochlea in **(A)**, especially the apical part of each turn, was smaller than the highlighted line, which was the outline of cochlea in T2 sequence in **(A′)**. Vestibule-grade I (four points) and semicircular canals-grade I (five points): because the black area in **(B)** is over one-third of the whole ampulla, shown in **(B′)**. Therefore, the sum EH level of this patient was 12 points.

3D-FLAIR MRI was performed on a 3-Tesla scanner (uMR 770, United Imaging, Shanghai, China) 24 h after intratympanic Gd injection with a 24-channel head coil. Three-dimensional heavily T2-weighted spectral attenuated inversion recovery (3D-T2-SPAIR, T2) and 3D-FLAIR imaging were subsequently performed. The main scan parameters for the 3D-FLAIR sequence were as follows: time of repetition (TR) = 6,500 ms, time of echo (TE) = 286.1 ms, time of inversion = 1,950 ms, scan time = 6 min and 11 s. The main scan parameters for 3D-T2-SPAIR sequence were as follows: TR = 1,300 ms, TE = 254.7 ms, scan time = 4 min and 30 s (12). The degree of EH of each part of the inner ear (include vestibule, cochlea, and semicircular canals) was assessed separately into four grades (Table 2), while VR scores were assigned and the EH were presented as the sum scores of each part according to our previous study (12) (Figure 2). The EH were estimated double-blinded by two experienced radiologists by which if there was any discrepancy, it would be double-checked by one senior otologist.

**Table 2:** Four gradings of the EH in the cochlea, vestibule, and semicircular canals.

**Grade**	**Cochlea**	**Vestibule**	**Semicircular canals (SCC)**
None	The scala media (dark area in the cochlea) could not be viewed.	The saccule and utricle (two dark areas in the vestibule) are separate; Saccule is smaller than the utricle.	The SCC is clearly visible; A narrow dark area (<1/3 of the ampulla) is visible.
Grade I	The scala media expand but still shapes as a triangle.	The saccule becomes larger than utricle; The saccule not confluent with utricle.	The SCC is clearly visible; The dark area occupies over one-third of the ampulla.
Grade II	The scala media expands into circle.	A confluence appears; Surrounding perilymph is still visible.	The ampulla becomes dark; Some of the SCC narrow or become invisible.
Grade III	No signal could be viewed in the scala media.	No signal could be viewed in the vestibule.	No signal could be viewed in the SCC.

*The grading algorithm is summarized from VR scores in previous He's work (12).

### Treatment

Different treatment protocols were administered variously for four hearing loss types according to the 2015 Chinese guideline (13). The main regimen for patients was intravenous dexamethasone. To achieve a better prognosis, hyperbaric oxygenation (16) and intratympanic dexamethasone injection (17) were used as the auxiliary therapies for patient. The treatment duration lasted for 10 days.

### Outcome Assessment

The therapeutic effect of UISSNHL is graded as follows (13): (1) Complete recovery: the follow-up audiometry returns to normal or reaches the healthy ear level or the level before the disease; (2) remarkable effect: the affected frequencies increase by more than 30 dB HL on average; (3) effective: the average hearing loss frequency rises by 15–30 dB HL; and (4) invalid: the mean improvement of affected frequencies is <15 dB HL. In this study, in order to facilitate statistical analysis, complete recovery or remarkable effect was collected as effective.

### Statistical Analysis

Descriptive statistics were done for age, sex, affected side, the incidence of vertigo, and PTA in UISSNHL patients. The nonparametric analysis and paired *t*-test were done for the two-group analysis while one-way ANOVA was used for four-group analysis. The logistic analysis was used to exclude the possible confounding factors. The possible relationships among EH, vertigo, and prognosis were compared by the Chi-square test and Fisher's exact test for categorical variables. *P* < 0.05 was statistically significant. Statistical analyses were conducted by using SPSS 22.0 for Windows software (IBM, Chicago, IL, USA).

## Results

### Clinical Characteristics of UISSNHL Patients

The clinical characteristics of the enrolled 72 patients and the distribution of the four hearing-loss groups are listed in Table 3. Among 72 UISSNHL ears, TD-G (31.9%) was the most common hearing loss type, LF-G (29.2%) and F-G (23.6%) were less common while HF-G (15.3%) consisted of the least of all. Vertigo manifested as a single rotational vertigo, swaying vertigo, which occurred 1 day before the hearing decline or after hearing decline and lasted from several hours to several days. The onset was not related to the head position, and the attack did not recur after recovery. The age of the four groups had no significant difference (*P* > 0.05). The patients with UISSNHL were divided into three groups according to their ages: <40 years old, 40–60 years old, and > 60years old. At the same time, EH of three groups were statistically analyzed by the Chi-square test, and no significant correlation was found (*p* > 0.05). Gender and affected sides were evenly distributed in each group (*P* > 0.05). Some patients reported a concomitant symptom of vertigo in each group in which TD-G had the highest rate. The average time from symptom onset to the first examination was 6.9 ± 4.4 days (1–20 days). The results of PTA before treatment and after treatment were described. The patients in LF-G had the lightest hearing loss level, while patients in TD-G had the most severe hearing loss. However, the highest effectiveness was in patients of LF-G (85.7%).

**Table 3:** Characteristics of UISSNHL patients.

	**LF-G**	**HF-G**	**F-G**	**TD-G**
Patients [*n* (%)]	21 (29.2)	11 (15.3)	17 (23.6)	23 (31.9)
Age (years)	54.1 ± 8.3	49.2 ± 11.3	50.1 ± 12.5	47.3 ± 8.6
**Gender (*****n*****)**				
Male	12	7	8	13
Female	9	4	9	10
**Affected side (*****n*****)**				
Right	13	8	6	8
Left	8	3	11	15
Vertigo [*n* (%)]	5 (23.4)	3 (27.2)	6 (35.3)	10 (43.5)
**PTA (dB HL)**				
Pretreatment	45.8 ± 5.2	51.7 ± 6.9	61.6 ± 8.4	98.9 ± 9.0
Post-treatment	21.2 ± 9.4	34.0 ± 9.8	49.5 ± 15.0	86.3 ± 16.7
Effectiveness [*n* (%)]	18 (85.8)	6 (54.5)	6 (35.3)	8 (34.8)
EH of <40 years old [*n* (%)]	1 (0.05)	1 (9.1)	0	0
EH of 40–60 years old [*n* (%)]	9 (42.3)	0	1 (5.9)	4 (17.4)
EH of >60 years old [n (%)]	1 (0.05)	1 (9.1)	1 (5.9)	0
Total EH [*n* (%)]	11 (52.4)	2 (18.2)	2 (11.8)	4 (17.4)

*n, means number*.

An analysis of the relationship between vertigo and hearing loss in four types is shown in Table 4, and there was no difference between vertigo and hearing loss types (*P* = 0.56).

**Table 4:** An analysis of the relationship between vertigo and hearing loss types.

**Vertigo or not**	**UISSNHL types (*****n*****)**			
	**LF-G**	**HF-G**	**F-G**	**TD-G**
Vertigo group	5	3	6	10
No vertigo group	16	8	11	13
*P*	0.56			

*The comparison was done between the “vertigo” group and the “no vertigo” group. There was no difference when comparing the “vertigo” group and the “no vertigo” group. The P-value was derived from the Chi-square test*.

### EH Evaluation and MRI Association With Clinical Characteristics

**Figure 3:**
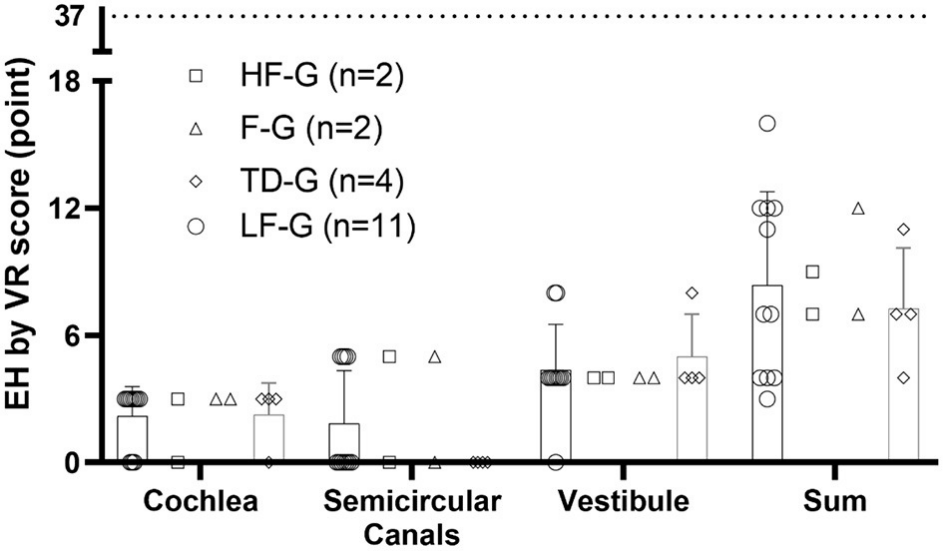
EH grading in three parts of the inner ear and the sum of VR scores. EH in cochlea were scored 0, 3, 6, and 9 for four grades while those in vestibule were scored 0, 4, 8, and 12 and those in the semicircular canals as 0, 5, 10, and 16 according to the volume-referencing scores (12). The sum score of three parts represented EH in the entire inner ear. The scatter plot showed the scores in each part of each patient, while the histogram presented for TD-G and LF-G represented mean and SD. In this study, most UISSNHL patients had mild or moderate EH as the maximum score of EH was 37 points.

No side effects such as tympanic membrane perforation, infection, and other complications after the intratympanic injection of Gd-DTPA were observed. The Gd-DTPA demonstration rate was 100% (72/72) in both the affected ears and the normal ears. The incidence of EH is shown in Table 1, and LF-G had the highest EH-positive rate by 52.4%. The EH rate of LF-G was higher than that of F-G and TD-G, and the difference was statistically significant (*P* = 0.009, *P*=0.014), respectively. Then, EH were estimated and the distribution of EH grading according to VR scores is exhibited in Figure 2. The total EH level was estimated by the sum score of three parts of the inner ear, namely, cochlea, vestibule, and semicircular canal. Of all EH-positive patients with UISSNHL, gradings of EH were mild or moderate hydrops (Figure 2) with the maximum score of EH as 37 points (12). No significant difference was found among HF-G, F-G, and TD-G in EH sum scores (*P* > 0.05). Cochlear EH and vestibular EH were more detectable than EH in semicircular canals in most patients (Figure 3).

There was no statistically significant relationship between EH with gender (*P* = 0.402) and age (*P* = 0.116) using logistic regression analysis. After judging the grading of each part in the inner ear and added into sum to represent the total EH level of each patient, no significant difference of the sum EH score was found in EH patients with four types (*P* = 0.081). Therefore, we separated patients into no EH group and EH-positive group for analysis. Since LF-G had the largest positive rate of EH, the related analyses were mainly done in this group.

The possible influence of the vertigo symptom on EH was analyzed (Table 5). The EH rate was higher in the vertigo group (67%) than that in the no vertigo group (50%). However, the no correlation between vertigo and EH in LF-G was found.

**Table 5:** An analysis of the relationship between vertigo and EH in LF-G.

**Vertigo or not**	**EH in LF-G**	
	**EH positive (*n*)**	**No EH (*n*)**
Vertigo group	2	1
No vertigo group	9	9
*P*	0.311	

*The comparison was done between the “vertigo” group and the “no vertigo” group. There was no difference when comparing the “vertigo” group and the “no vertigo” group. P-value was derived from Fisher's exact test*.

### The Prognosis Analysis

Analysis of the relationship between EH and prognosis of all groups and LF-G is shown in Table 6. The effectiveness rate of all groups with positive EH (63%) was higher than that of all groups without EH (49%). Furthermore, the effectiveness rate of LF-G with positive EH (82%) was lower than that of LF-G without EH (90%). However, there was no significant difference between EH-positive rate in the effective group and the invalid group both in all UISSNHL patients and in LF-G patients (*P* = 0.291, *P* = 0.414).

**Table 6:** An analysis of the relationship between EH and prognosis of all group and LF-G.

**EH**	**Prognosis of all groups**		**Prognosis of LF-G**	
	**Effectiveness (*n*)**	**Invalidation (*n*)**	**Effectiveness (*n*)**	**Invalidation (*n*)**
Positive	12	7	9	2
None	26	27	9	1
*p*	0.291		0.414	

*The comparison was done between the “positive” group and “none” groups. There was no difference when comparing the “positive” group and the “none” group. P-value was derived from the Chi-square test*.

The possible relationship of vertigo and prognosis was also analyzed. The effectiveness rate of LF-G with vertigo (60%) was lower than that of LF-G without vertigo (93%). However, no significant difference was found in the effectiveness rate between the vertigo group and the patients with no vertigo in LF-G (*P* = 0.128; Table 7).

**Table 7:** An analysis of the relationship between vertigo and prognosis in LF-G.

**Vertigo or not**	**Prognosis in LF-G**	
	**Effectiveness (*n*)**	**Invalidation (*n*)**
Vertigo group	3	2
No vertigo group	15	1
*P*	0.128	

*The comparison was done between the “vertigo” group and the “no vertigo” group. There was no difference when comparing the “vertigo” group and the “no vertigo” group. P-value was derived from Fisher's exact test*.

## Discussion

Our study confirmed that EH does appear in UISSNHL patients and had the closest correlation to the low-frequency hearing loss. Among the possible pathological mechanisms discussed in the guideline referencing the German guideline (18), the low-frequency hearing loss is supposed to be most likely correlated with cochlear or vestibular EH (11). Indeed, early in 1990, the possible relationship between SSNHL and EH was firstly studied on human temporal bone by Yoon et al. (19). They observed 4 of 11 temporal bones from eight patients with SSNHL, and the EH-positive rate for ISSNHL accordingly was 28.6%. Filipo et al. (20) reported greater SP/AP ratios in electrocochleography of ISSNHL patients with low frequency and flat audiometric profiles, suggesting a close relationship of EH and ISSNHL. The exploration of ISSNHL with EH almost stagnated until the visualization of EH with 3D-FLAIR was established (6). Duan et al. (21, 22) firstly demonstrated that MRI can clearly visualize inner ear imaging in animal studies. Subsequently, Nagawama et al. (5) were the first to mention the 3D-FLAIR sequences and firstly attempt EH imaging in ISSNHL patients (23). The low availability (faint enhancement) of EH imaging in ISSNHL patients 4 h after intravenous Gd reported by Nakashima's research group (23) might limit other researchers' interest until in 2017 they broke their own point of view (10). Given that few studies were done to explore the EH relationship with SSNHL, Horri et al. (24) reported that two of eight ISSNHL patients (excluding low-frequency SNHL) had EH in 2011 and Chen et al. (9) reported that EH exists in four of seven ISSNHL patients with vertigo in 2012. These researchers used intratympanic Gd which was claimed by Yamasaka et al. (25) by which intratympanic Gd provided stronger signals and had no remarkable side effect. The latest study about EH and ISSNHL was published in 2019 by Zheng et al. (11) who reported a difference of EH in affected ears and unaffected ears and considered EH in pantonal ISSNHL (analog to F-G and TD-G in this study) to be the secondary EH.

In the present study, the EH rate of total UISSNHL was 26.4% and only patients in LF-G had a higher rate of EH (52.4%). However, Okazaki et al. (10) observed EH positivity in 66% cochlea and 41% vestibule in ISSNHL (low-frequency SNHL patients were excluded). Their results varied a lot from low onset of EH in ISSNHL in three types (HF-G, F-G, and TD-G) in the present study. The high positive rate might be due to the scanning method they used, hydrops after 4 h intravenous Gd, which exhibited the EH images according to the reversed endolymph and perilymph signals (26). Acute labyrinth inflammation with protein exudate, or blood-labyrinth barrier (BLB) causing Gd diffusion in both endolymph and perilymph (27), could explain the low detection rate of EH in ISSNHL in three types in the present study using the endolymph imaging 3D-FLAIR sequence (28). Indeed, 3D-FLAIR scans were studied in the bleeding of ISSNHL 10 min after intravenous Gd injection in many studies (29–35), which might reflect the possible inflammation and BLB in the inner ear as firstly mentioned by Sugira et al. (29). Zhu et al. (33) did not recommend 4 h after intravenous Gd injection as a time point to image the inner ear in ISSNHL patients since there were no significant signal intensity changes between the images 10 min and 4 h after Gd injection using the 1.5-T MRI, in which EH were not discussed. Kim et al. (36) also showed no significant difference in signal intensity in the affected ears. However, Byun et al. (37) and Min et al. (38) suggested that the higher signal intensity observed in ISSNHL patients 4 h after Gd indicated a poor prognosis. Byun et al. (37) claimed that a higher signal intensity was shown in 4-h Gd-injection imaging than that in 10-min imaging. The increased signal intensity caused by increased permeability (due to BLB) suggested an increase in inner ear damage. The signal intensity in the inner ear after 24 h intratympanic Gd was never discussed because there might be a difference in round-window permeability (39). Therefore, 3D-FLAIR after intratympanic Gd may have limitations in observing the difference of signal intensity to predict the permeability of BLB and the prognosis of ISSNHL. Relatively, 3D-FLAIR after 4 h intravenous Gd might be more suitable for ISSNHL without LF-G.

**Figure 4:**
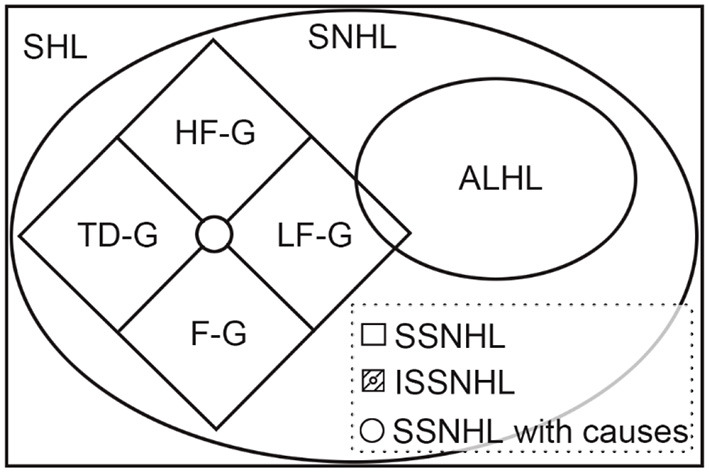
Subtype sets of the sudden hearing loss (SHL).

Interestingly, the recent Japanese researchers excluded the low-frequency hearing loss while analyzing ISSNHL, as mentioned above (10, 24). A nationwide epidemiological survey in Japan published in 2017 claimed that ISSNHL and acute low-tone sensorineural hearing loss (ALHL) belong to different inner ear diseases (40). ALHL is characterized by acute-onset low tone hearing loss often associated with tinnitus, ear fullness, and/or autophony, without vertigo, and its cause remains unknown (40). According to the 2019 AAO-HNS guideline and 2015 Chinese guideline, we considered there to be an intersection between ISSNHL and ALHL (Figure 4). ISSNHL patients with low-frequency hearing loss without vertigo were also ALHL and ALHL patients corresponding to the onset time and hearing loss definition which could be called ISSNHL. The LF-G in the present study might be divided into ISSNHL with vertigo and ALHL.

This figure shows the relationship among different acronyms related to sudden hearing loss. Among them, the definitions of SHL, SNHL, SSNHL, and ISSNHL were clearly explained in the 2019 AAO-HNS guideline (41) and the 2015 Chinese guideline (13). Sudden hearing loss (SHL) is defined as a rapid-onset subjective sensation of hearing impairment in one or both ears, while sensorineural hearing loss (SNHL) only means hearing loss without a conductive hearing loss (41). Furthermore, sudden SNHL (SSNHL) is a subset of SNHL developed within 72 h, which is the same in the 2019 AAO-HNS guideline (41) and the 2015 Chinese guideline (13). However, hearing loss definition varied in two guidelines. HF-G, LF-G, F-G, and TD-G were the abbreviations of four groups defined by the 2015 Chinese guideline (13). ALHL was defined according to the 2017 epidemiological survey in Japan. Although ALHL is separated from ISSHNL in the Japanese definition, there should be an overlap according to their definition.

Cochlear hydrops begins at the apical turn of the cochlea and extends to the vestibule, causing the low-frequency hearing loss ahead of vertigo (42). Shimono et al. (43) also reported that EH was observed in the cochlea and vestibule in ALHL. Furthermore, EH is a definite pathological feature and might be a cofactor of MD according to the comprehensive review from 1938 to 2012 (7). Ma et al. (44) conducted a comparison between ALHL and ISSNHL (low-frequency hearing loss excluded) and discovered that ALHL patients had higher IgE with an enhanced SP/AP ratio of electrocochleography, which was an index relating to EH in MD (12, 44). However, follow-up for ALHL patients was recommended because patients who presented with ALHL and concomitant tinnitus or had recurrent episodes of ALHL were more likely to develop MD than other ALHL patients (45). Junicho et al. (46) considered the ALHL as a subtype of ISSNHL. They reported that only 8.5% of 177 ALHL patients developed MD and concluded that not all low-tone ISSNHL patients suffered from EH even if they had vertigo attack at the onset. In summary, ALHL or ISSNHL patients with low frequency hearing loss might have EH in some patient and even develop MD with recurrent vertigo; however, the correlation was not definite. In our study, the 72 patients of ISSNHL ranged in age from 28 to 78. As the prevalence of MD is mostly between 40 and 60 years of age (47), the younger age group is less prone to be affected by MD. In order to reduce the bias, the patients with ISSNHL were divided into three groups (<40 years old, 40–60 years old, and >60 years old), and at the same time, EH of three groups were statistically analyzed by the Chi-square test, but no significant correlation was found (*p* > 0.05).

We concluded a similar result as the vertigo seemed to have no relationship for the EH-positive rate and the effectiveness in LF-G. Zheng et al. (11) had the same conclusion in 2009 and supposed that EH might be a secondary reaction of inner ear impairment. However, Yu et al. (48) made a META analysis in 2008 and found that ISSNHL patients with vertigo had a lower recovery rate of hearing than the ones without vertigo, while in the subgroup of these researches in which the patients were under the intratympanic corticosteroids injection, vertigo had no correlation with recovery rate of hearing. The intratympanic corticosteroids used as a treatment method might eliminate the prognostic difference. Also, the steroid–diuretic combination therapy was more effective than the steroid or diuretic treatments alone reported by Morita et al. (49), which also suggests a close correlation with EH and ALHL.

Consequently, 3D-FLAIR after 24 h intratympanic Gd showed 52.4% EH positivity in LF-G patients, and we recommended this scanning strategy for LF-G patients of ISSNHL. However, for the other three groups of patients, EH imaging was not recommended due to the low detection rate and no difference in effectiveness. Alternatively, 3D-FLAIR 10 min or 4 h after intravenous injection with the signal intensity assessment was recommended due to the possible BLB hypothesis.

This study has some limitations that should be highlighted. First, our ISSNHL patient sample size was considerably small after being divided into four groups. Second, the contrast MRI was not performed after treatment, which might show the EH changes and be helpful for understanding the possible etiology and pathogenesis of ISSNHL. Third, time delays between disease onset and MRI evaluation may be a confounder of the percentages of EH. At last, during our treatment and up to 1–2 months of follow-up of the patients, no patient ended having MD, but long-term follow-up results were not reported in this study.

## Data Availability Statement

The raw data supporting the conclusions of this article will be made available by the authors, without undue reservation.

## Ethics Statement

The studies involving human participants were reviewed and approved by Ethics Committee of Xinhua Hospital (Approval No. XHEC-D-2021-029). The patients/participants provided their written informed consent to participate in this study.

## Author Contributions

JY and MD contributed to the study design and critically reviewed and approved the final manuscript. HQ performed the data acquisition. HQ and BH contributed to the detailed study design and statistical analysis, interpretation of the results, drafting of the manuscript, and revision of the manuscript. HW and WW contributed to the study design, data acquisition, and statistical analysis. YL, JC, and FZ contributed to the methods of statistical analysis and critically reviewed the manuscript. All the authors contributed to the article and approved the submitted version.

## Conflict of Interest

The authors declare that the research was conducted in the absence of any commercial or financial relationships that could be construed as a potential conflict of interest.
